# Supplementary material for: Smoking and alcoholism dual addiction dissemination model analysis with optimal control theory and cost-effectiveness
Source: PLoS One. 2024 Oct 14;19(10):e0309356. doi: 10.1371/journal.pone.0309356 (PMC11472962; doi:10.1371/journal.pone.0309356)
Supplement: Attachment — Submitted filename: Review_report.pdf [file pone.0309356.s001.pdf]

Review report on the manuscript "***Modeling and Analysis of Smoking and Alcoholism Dual Addiction Dynamics with Optimal Control Theory and Cost-Effectiveness***"

I have carefully read the paper, and it seems interesting. Therefore, the manuscript can be considered further for publication in PLOS ONE if the authors are willing to incorporate a MAJOR Revision. Here under are my specific comments on the manuscript:

1. It is strongly advised and it is the responsibility of the authors to check the whole manuscript and correct all the grammatical errors and typos. The manuscript is full of grammatical errors and typos. It is enough to recommend rejection if the authors fail to thoroughly proofread their revised manuscript.
2. In the abstract, it is not clear on what control interventions were the optimal strategies and cost-effectiveness analysis carried on.
3. The last sentence in the abstract should be split into simple sentences with clear information.
4. There are a number of compound sentences with no clear meanings throughout the manuscript. For instance, see the first sentence under the introduction section. I suggest that the authors split this kind of sentences into simple ones with clear meanings.
5. There are a number of long sentences in the paper. For instance, the first sentence in the first paragraph on the second page is too long. The sentence should be split into simple and meaningful sentences.
6. The literature review should be updated with the following related works:
   a) https://doi.org/10.1007/s40314-022-01760-2
   b) https://www.lhscientificpublishing.com/Journals/articles/DOI-10.5890-JAND.2023.03.004.aspx
7. Description of the parameters delta_1, delta_2, delta_3, rho_1, rho_2 and rho_3 being "modification parameters" as mentioned by the authors in Table 1 is not clear. The physical meanings of the parameters should be given.
8. theta_1 and theta_2 should be described better than just being modification parameters. Please, see the recommended articles in (6) for similar parameter descriptions.
9. In the flowchart in Figure 1, the rate of progression from P_S to C_{SA} is missing. The authors should fix this.
10. The adjoint variables $f_i, i = 1,2,\ldots,11$ in Equation (21) and thereafter are different from their original definitions (with boldface). The authors should make corrections accordingly.
11. What is the meaning of (.) as appeared in the optimal state variables S, P and others in Theorem 9?
12. Remove the full stop (.) from the caption of all the figures.
13. Revise the captions of Figures 5 to 8 to reflect the specific strategy in terms of Strategy A to Strategy I.
