# Supplementary material for: Smoking and alcoholism dual addiction dissemination model analysis with optimal control theory and cost-effectiveness
Source: PLoS One. 2024 Oct 14;19(10):e0309356. doi: 10.1371/journal.pone.0309356 (PMC11472962; doi:10.1371/journal.pone.0309356)
Supplement: Attachment — Submitted filename: RESPONSETOREVIEWRCOMMENTS.docx [file pone.0309356.s002.docx]

Dear Editor, we have revised our manuscript “**Smoking and Alcoholism Dual Addiction Dissemination Model Analysis with Optimal Control Theory and Cost-Effectiveness**” accordingly.

**Editor Comments and Authors Responses**

1. Please ensure that your manuscript meets PLOS ONE's style requirements, including those for file naming. The PLOS ONE style templates can be found at
   <https://journals.plos.org/plosone/s/file?id=wjVg/PLOSOne-formatting> sample main body.pdf and [https://journals.plos.org/plosone/s/file?id=ba62/PLOSOne-formatting sample title authors affiliations.pdf](https://journals.plos.org/plosone/s/file?id=ba62/PLOSOne-formatting%20sample%20title%20authors%20affiliations.pdf)

**Response:** We have revised according to our previous experience in PLOS ONE Journal.

2. Please note that PLOS ONE has specific guidelines on code sharing for submissions in which author-generated code underpins the findings in the manuscript. In these cases, we expect all author-generated code to be made available without restrictions upon publication of the work. Please review our guidelines at https://journals.plos.org/plosone/s/materials-and-software-sharing#loc-sharing-code and ensure that your code is shared in a way that follows best practice and facilitates reproducibility and reuse.

**Response:** We agree with this comment

3. We note that your Data Availability Statement is currently as follows: All relevant data are within the manuscript and its Supporting Information files.

**Response:** Since we collected parameter values from published literatures and assumed values for illustration purposes, we mentioned “All relevant data are within the manuscript and its Supporting Information files” in the revised manuscript.

Please confirm at this time whether or not your submission contains all raw data required to replicate the results of your study. Authors must share the “minimal data set” for their submission. PLOS defines the minimal data set to consist of the data required to replicate all study findings reported in the article, as well as related metadata and methods (https://journals.plos.org/plosone/s/data-availability#loc-minimal-data-set-definition).

For example, authors should submit the following data:

- The values behind the means, standard deviations and other measures reported;
- The values used to build graphs;
- The points extracted from images for analysis.

Authors do not need to submit their entire data set if only a portion of the data was used in the reported study. If your submission does not contain these data, please either upload them as Supporting Information files or deposit them to a stable, public repository and provide us with the relevant URLs, DOIs, or accession numbers. For a list of recommended repositories, please see https://journals.plos.org/plosone/s/recommended-repositories.

If there are ethical or legal restrictions on sharing a de-identified data set, please explain them in detail (e.g., data contain potentially sensitive information, data are owned by a third-party organization, etc.) and who has imposed them (e.g., an ethics committee). Please also provide contact information for a data access committee, ethics committee, or other institutional body to which data requests may be sent. If data are owned by a third party, please indicate how others may request data access.

**Response:** All relevant data are within the manuscript and its Supporting Information files.

**Additional Editor Comments:**

1. The equation after the statement "The sum of all the differential equations described in equation (4)" should be on a new line and numbered.

**Response:** We have corrected accordingly.

2. Revise the abstract to contain the public health implications of the studies.

**Response: S**ince both smoking and alcohol addictions are related to health issues implementing the Strategy A has a great health implication regarding to reduce the dual addiction dissemination problem in the community. Accordingly we have revised the abstract.

3. Explain the terms in the basic reproduction number obtained, as done on page 6 of this paper https://doi.org/10.1016/j.heliyon.2023.e20531 after equation (5). Also, comment on the above paper since it is relevant to the current study. 

**Response:** We strongly agree with this strong comment and revised our manuscript accordingly by cited this up-to-date and related paper. See the last part of the sub-section 3.3.2.

4. What informed the authors' choice of the control terms since the authors did not perform a sensitivity analysis of the parameters in the R0.

**Response:** In analysis of our initial manuscript we performed sensitivity analysis but the total number of pages for the manuscript was more than 46 pages due to that we did not incorporated sensitivity analysis. Now since the editor comment 4 is very crucial in the revised manuscript based on the model parameters we have performed the sensitivity analysis together with its associated diagram and justified the corresponding optimal control problem**.**

**Reviewer #1: The study seeks to formulate a mathematical model to help mitigate the societal addictions to both smoking and alcohol intake. I find the work interesting if improved. These are my comments to the authors.**

1. The abstract of the study contains language construction errors. Also, the statement ". Finally, from the cost-effectiveness analyses we observed that implementing strategy is the most cost-effective strategy." in the abstract is not clear.

**Response:** We revised the abstract accordingly.

2. Authors should desist from citations of the form [24-32] where many papers are cited without explicitly explaining each of them and their results. There are several of them, kindly cite relevant articles with their key results well explained.

**Response:** We have restructured the literatures accordingly**.**

3. There are several grammatical errors in the work. Kindly read through the entire paper and correct them.

**Response:** We agree with this basic comment and we revised the full manuscript accordingly.

4. Please reconsider this statement "In the best of our understanding organized from a thorough literature review, no one is studied the smoking and alcoholism dual addiction dissemination in the community using mathematical modeling approach.” From my little search i came across this paper "Bhunu, C. P., & Mushayabasa, S. (2012). A theoretical analysis of smoking and alcoholism. Journal of Mathematical Modelling and Algorithms, 11, 387-408, which has extensively studied alcohol and smoking dual usage.

**Response:** We appreciate the reviewer search for similar studies what we have not addressed initially, however, after considering the reviewer comment we totally agree with the comment and searched to get similar studies in the same area thorough literature review and we could not find a study that have been constructed and analyzed the smoking and alcoholism dual addiction dynamics in the community with optimal control strategies and cost-effectiveness. Therefore, our study is different from the study suggested by the reviewer in different aspects.

5. Please change these phrases in the work "minimize and tackle".

**Response:** We have corrected accordingly.

6. Read the paper I have given out in point 4 and rewrite your contribution to literature. The novelty of the research is in doubt. Please clarify that. Why is the study not employing fractional calculus but integer order derivatives?

**Response:** We strongly agree and we have re-written the contribution and novelty of our study by comparing it with other similar studies like mentioned in comment 4. In our study we constructed the integer order model and in the near future any researcher can modify our study by considering fractional order approach to verify the effect of memory.

7. The section 2.1 to 2.3 headings are unnecessary. Let them all fall under section 2.

**Response:** We have ignore all the mentioned section headings accordingly.

8. Under model formulation, the compartment named as protection definition is ambiguous. Kindly explain that well and further established that from literature.

**Response:** The compartment $P\left( t \right)$ contains individuals who are protected against either smoking addiction (by education) or alcohol addiction (by education) or dual addiction denoted by$P(t)$. In similar approaches used in the studies of real world situations stated in [30, 31, 32] we considered education as the prevention mechanism.

9. Under model formulation, what do the authors want to communicate regarding the two compartments; addicted and permanently addicted? Please be clear with their definitions and support them with literature.

**Response:** We considered individuals who are addicted with smoking, but, can be improved (rehabilitated by taking treatment) denoted by$I_{S}(t)$, individuals who are permanently addicted with smoking throughout their life (i.e., could not be rehabilitated by taking any treatment measures) denoted by$P_{S}(t)$, individuals who are addicted with alcohol, but, can be improved (rehabilitated by taking treatment) denoted by$I_{A}(t)$, individuals who are permanently addicted with alcohol throughout their life (i.e., could not be rehabilitated by taking any treatment measures) denoted by$P_{A}(t)$. Since anxiety against mathematics is a serious social problem we refer for the case of permanent addiction from permanent anxiety from literature [29].

10. Kindly subject your assumptions to literature.

**Response:** Based on real world problem studies the studies stated in literatures [30, 31, 32] we have revised our model descriptions and assumptions accordingly.

11. Under schematic diagram, why should a permanently addicted individual (to alcohol or smoking) become a dual addicted before becoming a permanently dual addicted to both?

**Response:** We agree with this comment since both options are possible. In our model descriptions and assumptions we considered that individuals in the compartment $C_{SA}$ are dually addicted but they may be either smoking or alcohol permanent addicted. But, individuals in the compartment $P_{SA}$ are all permanently dual addicted with both smoking and alcohol. Thus, even though, there is a possibility where a permanently addicted individual (to alcohol or smoking) become a permanently dual addicted to both for the sake of simplicity we assumed that individuals who are either smoking or alcohol permanently addicted will be dually addicted before permanently dually addicted.

12. the model failed to consider an alcohol related or smoking related or even alcohol-smoking related death rates. Why?

**Response:** Initially we ignore death rate with addictions, however, after considering the reviewer comments we incorporated death rates due to illness related with addictions. Therefore, the revised manuscript considers different death rates due to illnesses related with addictions.

13. Authors should be explicit as in what it means that their model is positive and bounded.

**Response:** The model (4) solutions are both positive and bounded in the region $\Omega=\left\{ \begin{aligned} \left( S, P,E_{S},I_{S},P_{S},E_{A},I_{A},P_{A}, C_{SA},P_{SA}, T \right)\in\mathbb{R}_{+}^{11},N(t)\leq\frac{K}{\mu} \end{aligned} \right\}$ means it is invariant and attracting for dual addiction dynamical system (4). Thus, the dual addiction model is mathematically and epidemiologically well-posed and it is sufficient to consider the dynamics of the flow generated by the system (4) in Ω [28, 43].

14. Under the optimal control, authors should be specific with the efforts they are referring to. the controls look abstract in their current form.

**Response:** We have considered specific control measures such as education, punishment and psychological treatments.

15. Why is this "Characterization of Optimal Control" underlined? The paper has formatting problems. Kindly fix that.

**Response:** We have addressed this comment accordingly.

16. A number of the model parameters are assumed. Will the model be able to stand the test of time?

**Response:** In this study, to perform sensitivity analysis and to simulate the optimal control problem we adopted most of the parameter values from the available existing literatures, otherwise, we made suitable assumptions to carry out the simulations for illustration purposes.

17. The conclusion is too long. Paragraph one seems to be overelaborated. Other paragraphs should be simplified. Is there any literature support to your conclusions?

**Response:** We have revised the whole conclusion part very carefully.

**Reviewer #2: Review report on the manuscript “Modeling and Analysis of Smoking and Alcoholism Dual Addiction Dynamics with Optimal Control Theory and Cost-Effectiveness”**

I have carefully read the paper, and it seems interesting. Therefore, the manuscript can be considered further for publication in PLOS ONE if the authors are willing to incorporate a MAJOR Revision. Here under are my specific comments on the manuscript:

1. It is strongly advised and it is the responsibility of the authors to check the whole manuscript and correct all the grammatical errors and typos. The manuscript is full of grammatical errors and typos. It is enough to recommend rejection if the authors fail to thoroughly proofread their revised manuscript.

**Response:** We agree with this basic comment and we revised the full manuscript accordingly.

2. In the abstract, it is not clear on what control interventions were the optimal strategies and cost-effectiveness analysis carried on.

**Response:** The study carried out cost-effectiveness analysis to compare the cost incurred when we applied each of the proposed strategies A to I and in the revised manuscript abstract section we explained as “The cost-effectiveness analysis shows that implementing all the protection (education) control measures simultaneously (i.e. implementing Strategy A) is the most cost-effective strategy”

3. The last sentence in the abstract should be split into simple sentences with clear information.

**Response:** We re-wrote it again accordingly.

4. There are a number of compound sentences with no clear meanings throughout the manuscript. For instance, see the first sentence under the introduction section. I suggest that the authors split this kind of sentences into simple ones with clear meanings.

**Response:** We revised accordingly.

5. There are a number of long sentences in the paper. For instance, the first sentence in the first paragraph on the second page is too long. The sentence should be split into simple and meaningful sentences.

**Response:** We revised accordingly.

6. The literature review should be updated with the following related works:
a) https://doi.org/10.1007/s40314-022-01760-2
b) <https://www.lhscientificpublishing.com/Journals/articles/DOI-10.5890-JAND.2023.03.004.aspx>

**Response:** We have incorporated these up-to-date and related peer-reviewed published literatures to improve our revised manuscript.

7. Description of the parameters delta_1, delta_2, delta_3, rho_1, rho_2 and rho_3 being "modification parameters" as mentioned by the authors in Table 1 is not clear. The physical meanings of the parameters should be given.

**Response:** We have given the right physical meanings of all the mentioned parameters in the revised manuscript accordingly.

8. theta_1 and theta_2 should be described better than just being modification parameters. Please, see the recommended articles in (6) for similar parameter descriptions.

**Response:** We mean that $\theta_{1}$is the modification parameter that verify smoking and alcohol dual addicted individuals are more involved in the alcohol addiction dissemination process than the alcohol only addicted individuals. Similarly, we mean that $\theta_{2}$is the modification parameter that verify smoking and alcohol dual addicted individuals are more involved in the smoking addiction dissemination process than the smoking only addicted individuals.

9. In the flowchart in Figure 1, the rate of progression from P_S to C_SA is missing. The authors should fix this.

Response: Sorry for our mistake not to incorporate the term $\rho_{2}\lambda_{A}$ in the model flow chart what we have considered in the derivative of the state variables $P_{S}$ and $C_{SA}$ for the dynamical system (4). In the revised manuscript we have corrected this mistake.

10. The adjoint variables f_i,i=1,2,...,11 in Equation (21) and thereafter are different from their original definitions (with boldface). The authors should make corrections accordingly.

**Response:** We have corrected accordingly.

11. What is the meaning of (.) as appeared in the optimal state variables S, P and others in Theorem 9?

**Response:** We have re-written as$S^{*}(t), P^{*}(t)$,…. where $t>0$ is the time at the optimal state.

12. Remove the full stop (.) from the caption of all the figures.

**Response:** We have removed all the full stops accordingly.

13. Revise the captions of Figures 5 to 8 to reflect the specific strategy in terms of Strategy A to Strategy I.

**Response:** We have revised to reflect the impacts of the strategies A to I accordingly.
