# Supplementary material for: Smoking and alcoholism dual addiction dissemination model analysis with optimal control theory and cost-effectiveness
Source: PLoS One. 2024 Oct 14;19(10):e0309356. doi: 10.1371/journal.pone.0309356 (PMC11472962; doi:10.1371/journal.pone.0309356)
Supplement: Attachment — Submitted filename: RESPONSESFORSECONDREVISSION.docx [file pone.0309356.s003.docx]

Dear Editor, we the authors of the manuscript entitled “Smoking and Alcoholism Dual Addiction Dissemination Model Analysis with Optimal Control Theory and Cost-Effectiveness” thanks all the editors and reviewers for their great contribution in the review process and sharing knowledge of real world problem dynamics. Dear, Sir we have the following point by point responses.

**Editor and Reviewers Comments and Authors Responses**

**Editor Comments and Authors Responses**

The abstract and conclusion are well-written. However, the keywords should not contain words already in the title.

**Response:** According to the comment given above, we removed some word from the previous manuscript **and t**he keywords in the revised manuscript include: Smoking; Alcoholism; Smoking and Alcoholism Dual Addiction; Optimal Control Theory; Cost-Effectiveness, all are already in the title of the revised manuscript.

Other comments:

(1) The manuscript has a few grammatical errors and typos. The authors need to rectify these.

**Response:** According to the comment given to us we have read the whole manuscript again and again and corrected some grammatical errors we addressed in each page of the previous manuscript.

(2) Please clearly state the novelty of the work in connection to the mathematical modelling of the disease. What new compartment(s) have the authors added, and why do they need to add those compartment(s)?

**Response:** Dear editor, whenever we the authors studied different co-infection models on infectious diseases and we reviewed various published sources carried out by different scholars on the transmission dynamics of co-infections of infectious diseases. Also, in the formulation of this proposed dual addiction model we reviewed different co-existence models, however, to the best of the our understanding no one is formulated and analyzed eleven compartmental co-infection model by considering a protection compartment against both infections with optimal control theory and cost-effectiveness analysis. In comparisons with other scholars’ co-existence (dual existence) mathematical modeling studies on infectious diseases or social behaviors the new compartments that incorporated in our formulated model are the protection compartment denoted by $P$ and the compartment that contains individuals who are permanently dual addicted with both smoking and alcohol denoted by$P_{SA}$. We add these two compartments in our formulated model:

(i) To investigate the impacts of pre-exposure mechanism such as the impact of education protection mechanism on the dual addiction dissemination dynamics in the community.
(ii) To understand the smoking and alcoholism dual addicted individuals behavior, the dual addiction dissemination health impact, economic impact in the community and to verify how these addictions have long run impact on individuals who are permanently dual addicted with both smoking and alcohol ( since they cannot rehabilitated from their dual addiction).
(3) The authors should indicate in their motivation the new mathematical analysis used in studying this dynamics, which other researchers haven’t considered.

**Response:** From response of the comment 3 above we have explained that no alcohol addiction or smoking addiction or smoking and alcohol dual addiction mathematical model studies incorporated the protected group (protected compartment) against both addictions (by education), the permanently dual addicted group, optimal control theory and cost-effectiveness analysis in the dissemination dynamics of these addictions. Similarly, in case of mathematical analysis no smoking and alcoholism dual addiction mathematical modeling research studies analyzed the phenomenon of backward bifurcation.

(4) In the numerical section, the authors should improve on discussing the various Figures obtained and their impact on smoking and alcoholism control.

**Response:** We have improved the discussions by showing the impacts of the proposed control measures by comparing with other strategies in each case and the result determined without applying any control strategy.

**I kindly request you to provide a thoroughly revised manuscript along with a point-by-point response delineating how you addressed my general and specific comments; I meant exactly that. As to the point-by-point response, revised text in different colors is necessary, but that is not sufficient to let me know that you modified any given issue; it is necessary to answer specifically HOW you addressed/changed each issue in the response letter.

**Response:** We revised according to the editor suggestions.

**Reviewer 1 Comments and Authors Responses**

1. The paper still has some grammatical errors. Check the last statement in the abstract and also the entire paper.

**Response:** We have corrected some grammatical errors we addressed in each page of the previous manuscript.

2. The literature support for the assumptions should be cited before stating the assumptions.

**Response:** In page 5 of this revised manuscript, we made corrections and cited relevant studies for model assumptions before we started the assumptions.

3. Regarding my comment 8, your response indicated that similar works are done in [30-32], but you cited [38-40] instead in the article. Which one is the proper citation?

**Response:** Sorry for our mistake what we have done in the first revision process the according to the new reference arrangement in the first revision process the corrected citation should be [38-40]. Dear Sir, please consider the proper citation [38-41] for this revised manuscript.

4. The manuscript still has a lot of formatting inaccuracies. Please work on them.

**Response:** In the revised manuscript we have corrected various formatting inaccuracies
